# Controlling Shapes in a Coaxial Flow Focusing Microfluidic Device: Experiments and Theory

**DOI:** 10.3390/mi11010085

**Published:** 2020-01-13

**Authors:** Romen Rodriguez-Trujillo, Yu-Han Kim-Im, Aurora Hernandez-Machado

**Affiliations:** 1 Department of Electronic and Biomedical Engineering, University of Barcelona, Marti i Franques, 1, 08028 Barcelona, Spain; romen.rodriguez@ub.edu; 2 Department of Condensed Matter Physics, University of Barcelona, Marti i Franques, 1, 08028 Barcelona, Spain; yuhan.kim.im@gmail.com

**Keywords:** microfluidic, coaxial, interface, focusing, orientation, confocal

## Abstract

A coaxial flow focusing PDMS (polydimethylsiloxane) microfluidic device has been designed and manufactured by soft lithography in order to experimentally study a miscible inner flow. We studied a coaxially focused inner flow (formed by an aqueous fluorescein solution) which was fully isolated from all microchannel surfaces by an additional water outer flow. Different flow rates were used to produce a variety of flow ratios and a 3D reconstruction of the cross-section was performed using confocal microscope images. The results showed an elliptical section of the coaxially focused inner flow that changes in shape depending on the flow rate ratio applied. We have also developed a mathematical model that allows us to predict and control the geometry of the coaxially focused inner flow.

## 1. Introduction

The design and fabrication of microchips for cell and molecule handling has recently experienced a period of rapid development [1,2,3,4]. Among these microdevices, an important number have been fabricated to perform on-chip particle counting, analysis, and sorting. One particularly important application is the development of cell counters, which can be used as a stand-alone device for counting and sizing, or in more complex devices incorporating flow cytometry analysis for the discrimination and sorting of different cell populations (see Huh et al. 2005 [5] for a review on the subject).

One important feature of a microfluidic cell counter is that of accurate cell focusing in the cross-sectional area perpendicular to the direction of flow. Hydrodynamic focusing in the plane of the device is straightforward and has been used to study kinetics of chemical reactions [6] and to deliver chemicals with high spatial resolution [7]. Nevertheless, planar microchannel networks generally do not allow out-of-plane focusing. Therefore, particles in the focused stream are still spread in the out-of-plane direction [8]. Furthermore, if the flow is driven by pressure and has a parabolic profile (Poiseuille flow), their velocities are widely dispersed. This factor is a major complication for accurate flow cytometry in microfluidic devices [9]. The first microfluidic devices that achieved coaxial hydrodynamic flow focusing (both in-plane and out-of-plane directions) required laborious assembly [10] or operation [11]. 

For example, in refs [11] the authors demonstrated tight coaxial hydrodynamic flow focusing in a microfluidic device. They accomplished focusing of particles at the center of the cross-sectional area perpendicular to the flow and demonstrated a narrow velocity distribution of particles in the focused stream. However, experimental operation of the device results complicated with the need for accurate control of four or five independent flows at the time. 

In addition, some authors have implemented out-of-plane focusing that squeezes the sample flow towards the wall of the channel [12,13]. This approach is especially interesting when using wall integrated electrodes for sensing [14]; however it implies that the particles meet with the steep distribution of the characteristic parabolic profile and gives practical complications. 

Hebert et al. [15] improved the device presented by [11] obtaining coaxial flow focusing, but simplifying the experimental set-up by reducing the number of inlets to 3. They fabricated a micronozzle that permitted focusing the particle stream radially. Testa and co-workers developed a facile microfluidic platform with three inlets to perform 3D-microflow cytometry with hydrodynamic focusing and integrated optical fibers [16]. In a similar way, Chiu et al. [17] fabricated a 3D microstructure with a nozzle in the fluid junction that also enabled coaxial flow focusing. They demonstrated good performance for particle detection using fluorescence and reported a method for measuring velocity of individual particles. A different approach has been presented by Lin et al. [18] with a very simple fabrication method with a single channel layer and single sheath flow inlet. They tested the device for a variety of total flow rates and flow rate ratios, obtaining coaxial flow focusing for some of them. More recently, Liu et al. [19] presented coaxial flow focusing with high precision until the sub-micrometer range by fabricating a monolithic integrated glass micronozzle. This device was useful to study the dynamics of mixing at very small scales.

We propose here a simple microfluidic device that is able to perform coaxial hydrodynamic flow focusing with a very simple fabrication method. The device is very straightforward to use because it only requires a sample flow and a single sheath flow. We studied experimentally the behavior of the fluids and demonstrated accurate control of the shape of the inner focused flow. We reconstructed the 3-dimensional structure of the system with a confocal microscope. A mathematical model has been established which permits the prediction of the shape of the focused stream in an accurate manner. This device could be used for accurate single cell focusing to be implemented in flow cytometry. The developed mathematical model permits determination of the conditions to control the shape of the inner flow with a cross-section that can be tuned from circular to elliptical. This property of our system is especially helpful to accommodate cells with distinct shapes, as is the case of red blood cells (discocytes).

## 2. Materials and Methods 

**Figure 1:**
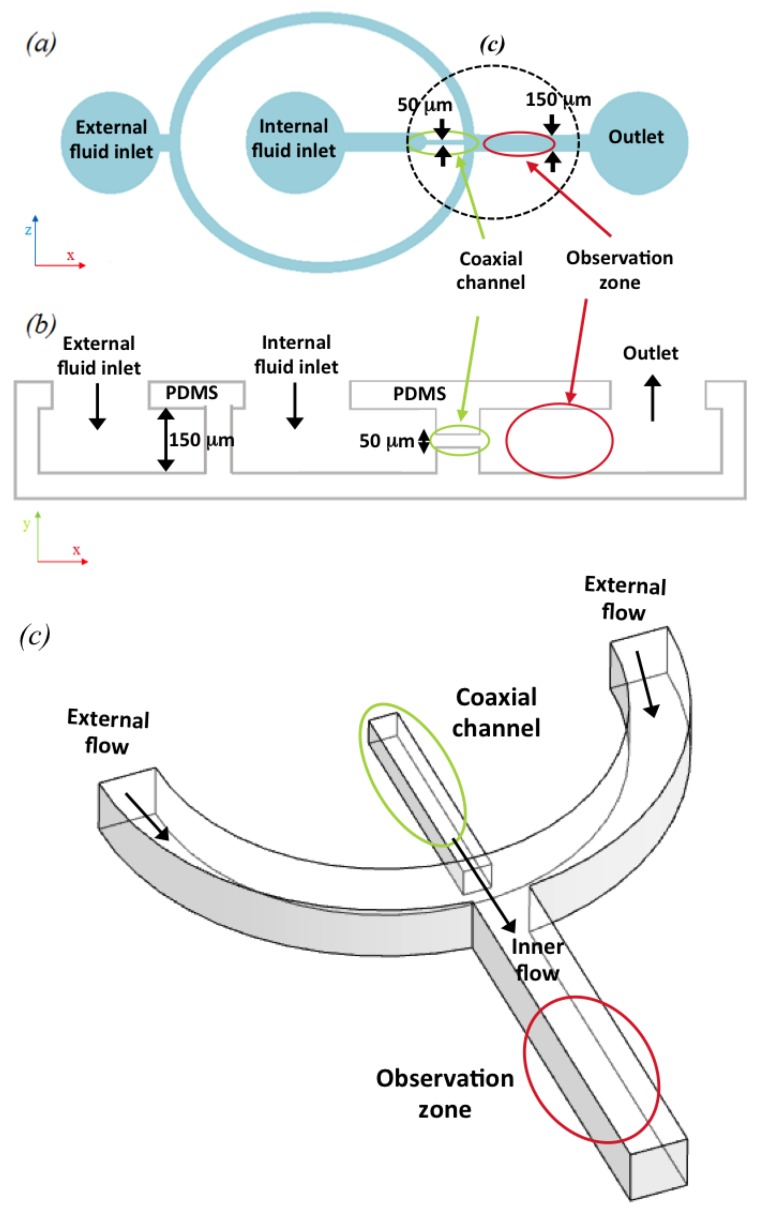
Design of the coaxial flow focusing microfluidic device: (**a**) Representation of a general (*x*,*z*)-plane view of the design; (**b**) longitudinal section of the device (*y*,*z*). The coaxial microchannel is circled in green, the observation zone, where the inner flow is formed, is marked in red; (**c**) A 3D amplification of the junction where the external flow and inner flow are combined (marked in black in Figure 1a). The coaxial inner flow is represented with a green cylinder. The diagrams are not to scale.

The experimental set-up consisted of the microfluidic device (Figure 1), two syringe pumps with different flow rates, and a confocal microscope with a coupled CCD camera.

**Figure 2:**
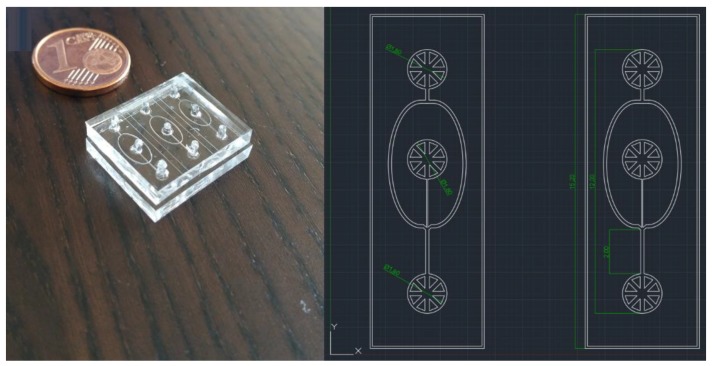
(**left**) Image of the polydimethylsiloxane (PDMS) microfluidic device structure once aligned and bounded. There are a total of three coaxial systems in a single piece of PDMS, which is similar to the size of a one cent coin. (**right**) AutoCAD design of the microfluidic system with representative dimensions shown in mm. The region where the coaxial inner flow is formed has 2 mm of length.

A soft lithography fabrication technique, previously described by Abate et al. [20], was used to produce two PDMS (polydimethylsiloxane) constructs. A coaxial microchannel microfluidic device was then produced by carefully aligning and bonding the two pieces of PDMS, leaving a microchannel system between the inner surfaces of the PDMS structures. The two PDMS pieces had a channel pattern corresponding to this microfluidic device which was designed specifically for this work (see Figure 2, right). Bonding of the PDMS structures was achieved using a different method. The patterned surfaces were plasma treated with an O_2_ plasma corona discharge (Corona SB, Black Hole Lab) before contact bonding.

Based on this previous work [20], alignment of the PDMS pieces was achieved by creating additional structures on the surfaces to form a mechanical lock. When the PDMS structures slide across each other, protruding structures on one surface fit into recesses in the other surface allowing an easy assembly and as a result, obtaining the final and correctly aligned microfluidic device. 

The final microfluidic device was connected with one syringe pump at a constant flow rate of 60 mL/min for the external flow while the other pump used a varying flow rate for the internal flow between 0 and 120 mL/min. This generated the range of flow rate ratios used in this work. The fluids used in this study were miscible. Water was used for the external flow and an aqueous solution of fluorescein (0.05 mg/mL, Sigma–Aldrich, St. Louis, MO, USA) was used for the inner flow.

## 3. Results

### 3.1. Mathematical Model

**Figure 3:**
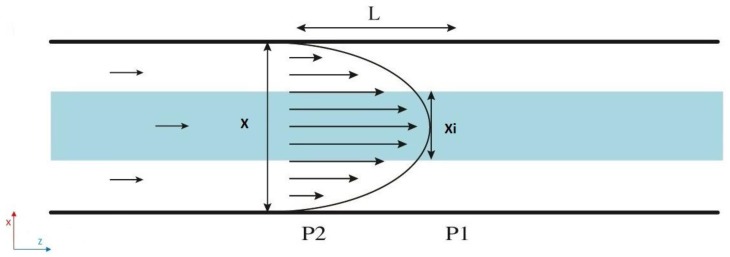
Representation of the Poiseuille velocity profile of our microfluidic system in the (*x*,*z*)-plane. The inner flow is represented in blue and the outer flow in white. A single parabolic profile is the result of the miscibility of the fluids. As consequence, the pressure drop from point P2 to P1 of the total flow (inner and outer flow) and the pressure drop in the inner flow from P2 to P1 are the same.

In this study, the inner profile is observed by an elliptical cross-section with two independent axes. In the following, we propose a mathematical model to predict this inner flow cross-sectional shape. A water-based solution of fluorescein and pure water were used for the inner and outer flow respectively. Given that the two fluids are miscible, the Poiseuille velocity of the entire complex system should follow a parabolic profile (see Figure 3).

The pressure drop over distance L downstream for the inner flow can be simply expressed as [2]:

(1) $$\Delta P=\frac{64\mu L\left(1+{\left(\frac{x_{i}}{y_{i}}\right)}^{2}\right)}{x_{i}{}^{3}y_{i}\pi }Q_{int}$$

taking into account that the flow has an elliptical cross-sectional shape ($S_{ellipse}=\frac{\pi }{4}x_{i}y_{i}$), where *µ* is the dynamic viscosity of the fluid, *Q_int_* is the applied flow rate for the inner flow, x_i_ is the minor diameter, and *y_i_* the major diameter.

The physical microchannel has a square cross section. Therefore, we can define the pressure drop for the total flow over distance L as a function of a square section ($S_{square}=X^{2}$) as [2]:

(2) $$\Delta P=\frac{28.3\mu L}{X^{4}}Q_{t}$$

where Q_t_ is the total flow rate applied to the whole system (*Q_t_* = *Q_int_ + Q_ext_*), and X is the side of the square section of our microchannel. 

Since the pressure drop for the total flow and the inner flow are the same, Equations (1) and (2) should be equal and we obtain a function of *x_i_* and *y_i_* that depends on the flow rate ratio (*Q_int_/Q_t_*):

(3) $$f\left(x_{i},y_{i}\right)=\frac{x_{i}{}^{3}y_{i}}{1+{\left(\frac{x_{i}}{y_{i}}\right)}^{2}}=m_{th}\frac{Q_{int}}{Q_{t}}$$

This equation shows a linear behavior of f(*x_i_*,*y_i_*) as a function of the flow rate ratio *Q_int_/Q_t_* with a predicted slope of *m_th_* = 3.64 × 10^8^ μm^4^. This value should be compared with the one obtained from experimental results.

### 3.2. Experimental Results

**Figure 4:**
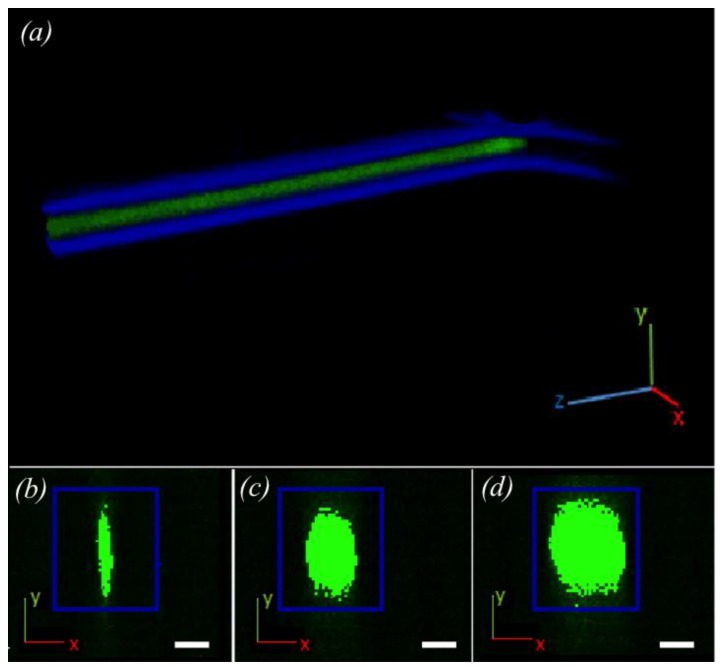
Top: (**a**) an example of 3D reconstruction with ImageJ software from the confocal microscope images. The blue feature corresponds to the walls of the microchannel where the coaxial inner flow (*Q_int_*) is formed, which is represented in green. Bottom: (**b**–**d**) There are three different examples of reconstructed cross sections (*x*,*y*-plane) of the inner flow at different flow ratios (b: 5 mL/min, c: 30 mL/min, d: 75 mL/min). Scale bar: 50 μm.

From the CCD camera coupled to the confocal microscope, images were obtained from the fluorescence produced by the analyte within the device. Several images were taken for each flow condition from which a 3D reconstruction of the coaxial focused inner flow was produced (Figure 4a). This technique allowed us to visualize the cross section form of the inner flow (Figure 4b–d) and to simply characterize it as a linear function of the flow rate ratio.

Using the described apparatus, images obtained from the confocal microscope were used to produce a 3D reconstruction of the fluorescence of the coaxial inner flow (see Figure 4). This reconstruction verified that the inner florescent flow was fully surrounded by the non-fluorescent external flow. In order to produce a clear image of this phenomenon, we delimited an observation zone (see Figure 1, red circle) where the inner flow showed a stable behavior. This zone was after the point where the external flow compressed the internal flow coaxially but before diffusion and other interference concealed the coaxially focused inner flow effect. The shape of the section was analyzed to determine the geometrical form of the inner flow. However, this form was not constant but changed as the flow rate ratio increased. 

In Figure 4, reconstructions from the results of different conditions used in the experiment can be seen which showed different cross sectional shapes of the coaxial inner flow. The images were analyzed and the inner flow diameters in both axes were measured using ImageJ. Under conditions where the external flow rate was significantly larger than the internal, the coaxial inner flow formed an elliptical shape. For higher internal flow rates, the coaxial inner flow tended to form an approximately circular shape. The inner flow did not increase the distance for both axes equally but it grew faster in the x direction than for the y-axis. 

**Figure 5:**
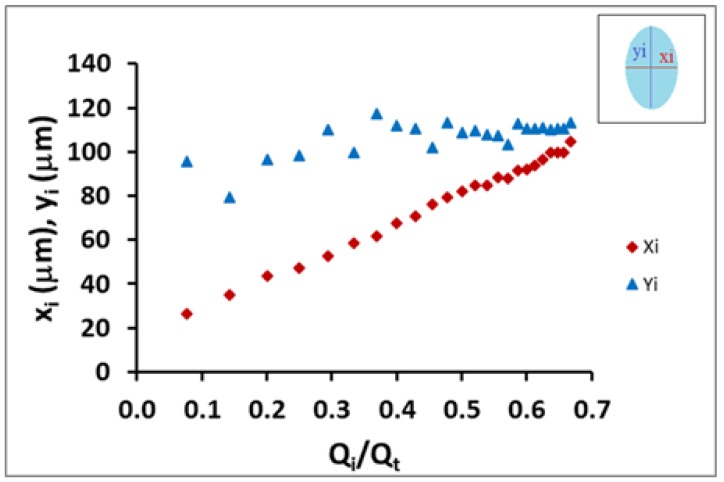
Plots of the behavior of the minor axis of the coaxial ellipse jet (*x_i_*) and major axis (*y_i_*) versus the flow rate ratio (*Q_int_/Q_t_* where *Q_t_ = Q_int_ + Q_ext_*), shown as blue triangles and red diamonds respectively. The growth of both axes is not the same as the flow rate ratio is increased. The aspect ratio *x_i_*/*y_i_* of the inner flow varies by controlling *Q_int_* for a fixed *Q_ext_*. It is possible to change from an ellipse cross-section to a circle by increasing *Q_int_*.

In Figure 5, we show the behavior of the minor axis of the coaxial ellipse jet (*x_i_*) and major axis (*y_i_*) versus the flow rate ratio. From the data obtained, the difference in changing inner flow cross-section shape could be quantitatively observed. While the minor-diameter (*x_i_*) of the initial elliptical cross-section shape increases rapidly, the major-diameter (*y_i_*) showed a slower increase; however, this was significant and could not be considered to be constant. Moreover, both diameters were mathematically related to each other and the flow rate ratio. Therefore, the mathematical model developed in the previous section allowed us to better characterize the geometrical shape of the coaxial inner flow (Equation (3)).

**Figure 6:**
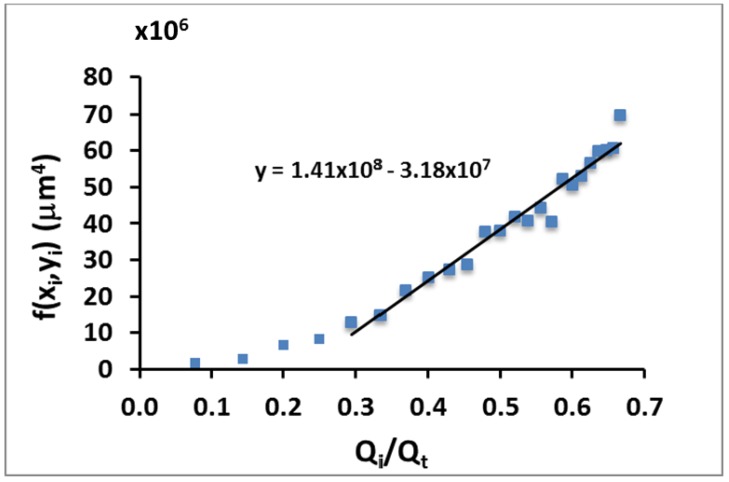
Plot of function f(*x_i_*, *y_i_*) defined in Equation (3) for different values of *Q_i_/Q_t_*. The values of x_i_ and y_i_ are experimental measurements. The continuous line is the regression line of the data with a slope *m_exp_* = 1.41 × 10^8^ μm^4^ with an *R^2^* value of 9.55. The theoretical model predicted a slope of *m_th_* = 3.64 × 10^8^ μm^4^.

Using the experimental data measured from confocal microscopy images, the function f(*x_i_*,*y_i_*) versus the flow rate ratio has been also plotted (Figure 6). This experimental data shows a linear tendency, as expected from the theoretical model, with a slope of *m_exp_* = 1.41 × 10^8^ μm^4^ (Figure 6). This value is slightly smaller from the one obtained from the theory (*m_th_* = 3.64 × 10^8^ μm^4^ directly derived from Equation (3)) The difference between the theoretical and experimental slope comes from the behavior of the system in the lower regime (*Q_int_* << *Q_t_*), where the height *y_i_* of the focused fluid is not accurately defined (see the highly dispersed data presented in Figure 5 for this lower regime). In this regime where the sample focused flow rate is small compared to the focusing fluids, the stability of the flow is compromised and then, the condition of elliptical cross-section is disturbed. That makes the correct measurement of the ellipse dimensions impractical and thus gives rise to a considerably big error, which explains the discrepancy between experimental and theoretical data.

## 4. Discussion

The primary objective of this work was to study the interaction between two flows within a self-manufactured coaxial flow focusing microfluidic device designed specifically for this purpose. Microfabrication using a soft lithography technique has been widely applied in previous studies with more or less laborious fabrication schemes or complicated experimental set-ups [15,16,17,18,19]. However, in this work, a coaxial flow focusing microchannel has been developed using a simple planar lithography technique with additional locking structures to perform correct alignments. This fabrication process for producing a coaxial flow-focusing device is not new, as it has been used before to produce double emulsions [20]. We have used it here instead to show a different interesting application. Indeed, this new device allowed the observation of a phenomenon in which a fully isolated elliptical inner flow was coaxially focused by an aqueous liquid outer flow.

The shape of the inner flow was characterized by using confocal microscopy. The particular geometry of our microfluidic device at the region of fluid intersection resulted in an inner flow, which was fully surrounded by an external coaxial focusing flow. The way the flow was pinched laterally produced an anisotropic pressure, which derived in an elliptical shape of the perpendicular section. As the two flows were fully miscible, there was no surface tension and therefore the inner flow kept this shape throughout the entire length of the microchannel.

In addition, the eccentricity of the ellipse could be easily controlled by varying the inner and the outer flow rates. The fact that the inner flow changes its perpendicular shape could easily contribute to the current techniques for separating and trapping particles and cells.

As we have already stated throughout the text, studying the generation of a miscible co-flow has been extensively presented before [6,7,8,9,10,11,12,13,14,15,16,17,18,19] and, especially, high-quality studies on the conditions for flow stability have been performed [21]. In our work, we have gone further by contributing a theoretical framework that explain and predicts the stability conditions between two miscible fluids in a coaxial flow-focusing device.

A feature that could be improved in our system is that, due to the way the flow is pinched laterally, the shape of the focused flow is limited to a vertical ellipse. In order to solve this, the focusing flow should also pinch the sample flow vertically to have a coaxially distributed pressure.
